# Kinetic Modeling, Thermodynamic Approach and Molecular Dynamics Simulation of Thermal Inactivation of Lipases from *Burkholderia cepacia* and *Rhizomucor miehei*

**DOI:** 10.3390/ijms23126828

**Published:** 2022-06-19

**Authors:** Natividad Ortega, Laura Sáez, David Palacios, María D. Busto

**Affiliations:** Department of Biotechnology and Food Science, Area of Biochemistry and Molecular Biology, University of Burgos, Plaza Misael Bañuelos s/n, 09001 Burgos, Spain; nortega@ubu.es (N.O.); lsm0001@alu.ubu.es (L.S.); dpalacios@ubu.es (D.P.)

**Keywords:** lipases, thermal inactivation, thermodynamic parameters, molecular dynamics simulations, *B. cepacia*, *R. miehei*

## Abstract

The behavior against temperature and thermal stability of enzymes is a topic of importance for industrial biocatalysis. This study focuses on the kinetics and thermodynamics of the thermal inactivation of Lipase PS from *B. cepacia* and Palatase from *R. miehei*. Thermal inactivation was investigated using eight inactivation models at a temperature range of 40–70 °C. Kinetic modeling showed that the first-order model and Weibull distribution were the best equations to describe the residual activity of Lipase PS and Palatase, respectively. The results obtained from the kinetic parameters, decimal reduction time (D and t_R_), and temperature required (z and z’) indicated a higher thermal stability of Lipase PS compared to Palatase. The activation energy values (Ea) also indicated that higher energy was required to denature bacterial (34.8 kJ mol^−1^) than fungal (23.3 kJ mol^−1^) lipase. The thermodynamic inactivation parameters, Gibbs free energy (ΔG^#^), entropy (ΔS^#^), and enthalpy (ΔH^#^) were also determined. The results showed a ΔG^#^ for Palatase (86.0–92.1 kJ mol^−1^) lower than for Lipase PS (98.6–104.9 kJ mol^−1^), and a negative entropic and positive enthalpic contribution for both lipases. A comparative molecular dynamics simulation and structural analysis at 40 °C and 70 °C were also performed.

## 1. Introduction

Lipases (triacylglycerol ester hydrolases EC 3.1.1.3) are widely applied in different industrial sectors such as food, dairy, flavors, pharmaceutical, biofuels, leather, cosmetics, detergents, and chemicals [1]. Lipases are of plant, animal, and microbial origin, but microbial lipases are produced at the industrial level and represent the most widely used class of enzymes in biotechnological applications and organic chemistry [2].

Most lipases have a helical motif, known as “lid”, that covers the catalytic center and controls the substrate access to the active site. This dynamic domain is very likely to affect both catalytic properties as well as the stability of the biocatalyst. Based on the active center and lid domain, Gutiérrez-Ayesta et al. have identified three types of lipases: (i) lipases with an active site and a lid on the surface of the enzymes (*Rhizomocur* family), (ii) an active site and a funnel-like lid (*Pseudomonas* and *Candida antarctica* family), and (iii) a very small lid and a funnel-like binding site (*Candida rugosa* family) [3]. Lipases from *Burkholderia cepacia* and *Rhizomucor miehei* are included in the (ii) and (i) group, respectively.

The main factors that contribute to the versatility of lipases are their ability to catalyze hydrolysis reactions in aqueous media and synthesis reactions in non-aqueous media, and their high substrate specificity (regio-, enantio-, and chemo-specificity) [4]. Next to selectivity and activity, thermostability is one of the most desirable traits of lipases [5,6]. Several structural factors define this property such as the number of hydrogen bonds, salt bridges, stabilization of secondary structures, occurrence of disulfide bonds, higher number of proline residues, higher polar surface area, hydrophobicity, shortening of loops, and stabilization of the lid domain [3,7].

It has been widely demonstrated that thermal stability and heat inactivation studies are an important step for the characterization of commercial lipases from both scientific and industrial perspectives [8,9]. The thermal stability of enzymes can be studied by various approaches: assay of residual enzyme activity against time, determination of changes in protein conformation by circular dichroism, fluorescence, or other techniques [10]. Among them, one of the most used is the determination of the half-life time at a temperature since it provides useful information for the industrial applications of the biocatalysts. Various behaviors have been found when studying the kinetics of enzyme deactivation, some enzymes follow an exponential decrease in activity over time, while others exhibit a non-exponential behavior [11]. In fact, the mechanism of the thermal deactivation of the enzymes has been explained by several models [12,13,14]. Modeling the kinetics of thermal decomposition can provide a better understanding of the functionality of the enzyme allowing the development of bioprocesses with greater efficiency.

Kinetic and thermodynamic studies of the thermal inactivation of lipases from different sources have been described [15,16,17,18]. Nevertheless, investigations on the thermal deactivation of industrial lipases from *R. miehei* and from *B. cepacia* are scarce [19,20,21,22].

Based on the above context, the present study evaluates the thermal stability data obtained for two commercial lipases, Lipase PS from *B. cepacia* and Palatase from *R. miehei*. Various mathematical models proposed to predict lipase residual activity as a function of time were statistically analyzed. Further characterization of both lipases was carried out with respect to thermodynamic behavior under varying temperatures. In addition, the influence of the temperature is described through molecular dynamics (MD) simulation and structural analysis.

## 2. Materials and Methods

### 2.1. Materials

Lipases were purchased from Sigma-Aldrich Corporation (St. Louis, Mo, USA): Lipase PS from *B. cepacia* (Amano) and Palatase from *R. miehei* (Novozymes). Gum arabic, *p*NP-palmitate (*p*NPP), and *p*-nitrophenol (*p*NP) were purchased from Sigma-Aldrich Corporation. All other chemicals used in the present study were of analytical or better grade without further purification.

### 2.2. Lipase Assay

Lipase activity was evaluated according to Palacios et al. [23], using pNPP as substrate. The reaction mixture consisted of 3.5 mL of 50 mM Tris-HCl buffer (pH 8.0) containing 1 g L^−1^ gum arabic and 0.4 mL of 15 mM pNPP dissolved in 2-propanol. The mixture was prewarmed at 40 °C, and then 0.1 mL of enzyme solution was added. After 5 min of incubation at 40 °C, the reaction was stopped by adding 1.5 mL of chloroform:isoamyl alcohol (24:1). The samples were centrifuged at 10,000 rpm for 5 min at 4 °C and the optical density of supernatant was measured at 410 nm using a spectrophotometer (Hitachi U-2000). In parallel, a calibration curve using pNP in 2-propanol (ranging from 0 to 180 mM) treated with chloroform:isoamyl alcohol (24:1) was run. Controls in which the enzyme solution was substituted by 50 mM Tris-HCl buffer (pH 8.0) containing 1 g L^−1^ gum arabic were included. One unit of activity (U) was defined as the amount of enzyme that liberates 1 μmol of pNP per minute under the assay conditions.

### 2.3. Thermal Inactivation

Thermal inactivation was carried out at four temperatures 40, 50, 60, and 70 °C. The samples of lipases were incubated at a selected temperature and withdrawn at time intervals ranging from 2 to 240 min. Immediately, the samples were placed in ice to stop thermal inactivation. Afterwards, the residual lipase activity was measured. All experiments were repeated three times.

### 2.4. Kinetic Models of Enzyme Inactivation

Several kinetic models describe the thermal inactivation of the enzymes, based on the diversity of phenomena involved in this process (chemical decomposition, aggregation, dissociation, denaturation, and coagulation) [24]. In this paper, the analysis of the experimental thermal inactivation data of lipases from *B. cepacia* (Lipase PS) and *R. miehei* (Palatase) was carried out by testing eight different kinetic models, applying the inactivation equations (Equations (1)–(8)) described in Table 1.

The thermal inactivation of enzymes has been usually explained by a first-order kinetic (Equation (1)) [12,15,16,18,25], which assumes the existence of a unique single enzyme. The Weibull distribution model (Equation (2)) considers that, under the experimental conditions, the momentary rate of thermal sensitivity to heat depends on the residual activity and the transient heating intensity, but not on the rate at which the residual activity has been reached [32]. The Weibull model is characterized by the parameters *n* (shape factor) and *b* (scale factor), which determine, respectively, the shape of the distribution curve and its scaling [26]. Equations (3)–(6) (Table 1) describe models that suggest the existence of a mixture of enzymes with different catalytic properties and/or heat sensitivities, that their residual activity can be described by the sum of two exponential decays, one for the expected heat-labile enzyme and the other for the stable. In the distinct isoenzymes model (Equation (3)), A_L_ and A_S_ represent the residual activities for the “labile” and “stable” isoenzymes, respectively, and *k*_L_ and *k*_R_ are the first-order reaction rate constants for each fraction [12,27]. The two-fraction model [27,28], where the coefficient *a* represents the active fraction of the thermal labile group in relation to the total activity, is detailed in Equation (4). The characteristics of a multi-component first-order model (Equation (5)) were discussed by Fujikawa and Itoh [29]. This model explains the inactivation of isoenzymes with different thermal stability and is represented as the sum of the thermal inactivation kinetics of their individual components. The series-type model is based on a succession of two irreversible first-order steps: an irreversible conversion of the native enzyme (E) to an intermediate (E_1_) with lower specific activity and the irreversible inactivation of E_1_ to an inactive enzyme (E_D_) [30]. In this model, the residual activity is described by Equation (6) (Table 1). Equation (7) describes an *n*th order decay model for thermal inactivation of enzymes (*n* is the order of the reaction) [28]. Fractional conversion (Equation (8)) describes a first-order inactivation process where the presence of a highly heat-resistant fraction (*A_r_*) prevents the total inactivation of the enzyme upon prolonged heating [31].

### 2.5. Statistical Analysis and Model Evaluation

The experimental data obtained in the thermal inactivation of commercial lipases were fitted to modeled theoretical curves and the kinetic parameters were determined using a non-linear regression analysis by SPSS Statistics 24 statistical analysis system software package.

Physical and statistical criteria were considered to establish the best fit of the models studied. Physical criterion is the absence of negative estimated parameters [33]. The statistical criteria include the coefficient of determination (r^2^), chi-square (χ^2^) (Equation (9)), and the standard error of means (*SEM*) (Equation (10)).

(9) $$\chi^{2}=\frac{\sum {\left(y_{exp}-y_{pred}\right)}^{2}}{\left(m-p\right)}$$

(10) $$SEM=\frac{\sum {\left(y_{exp}-y_{pred}\right)}^{2}}{\sqrt{m}}$$

where *m* is the number of observations, and *p* is the number of parameters. *y_exp_* and *y_pred_* are the experimental and predicted residual enzyme activities, respectively. The model with the lowest χ^2^ and *SEM*, and higher r^2^ for the residual activity is the best choice from a statistical point of view.

### 2.6. Molecular Dynamics Simulations

The crystal structures used in MD simulations, with PDB codes 3TGL (lipase from *R. miehei*) [34], and 3LIP (lipase from *B. cepacia*) [35], were obtained from the Protein Data Bank (PDB) database (http://www.rscd.org/pdb/ accessed on 8 April 2022). Proteins were solvated in a cubic box using the TIP3P water model, with the protein located at least 10 Å from the box edges, and sodium chloride at 0.15 mol/L was added in order to neutralize the net charge of the system [36]. The dimensions of the solvation box were 77 and 86 Å in each direction (x, y, z) for 3TGL and 3LIP, respectively. The simulated system comprised 46,807 atoms for Palatase and 64,141 atoms for Lipase PS, including hydrogens.

All systems were subjected to minimization using the conjugate gradient method. The MD protocols of equilibration and production were performed with the NAMD software via the NAMD graphical interface using the CHARMM36 forcefield in the NVT ensemble for all runs [36,37]. The overall MD procedure was aimed at modeling the thermostability of both lipase structures setting the temperature at 313 K and 343 K using Langevin dynamics with 50 ns of simulation time for the production runs. A distance cut-off of 12.0 Å was applied to short-range, non-bonded interactions, and 10.0 Å for the smothering functions. Long-range electrostatic interactions were treated using the particle-mesh Ewald (PME) method [38]. The analysis of the MD simulations for production runs was performed using the analysis tools and Tcl scripts implemented in VMD software [39].

## 3. Results and Discussion

One of the primary requirements for an enzyme to be of industrial importance is its functional stability, which is guided by both kinetic and thermodynamic parameters [40]. Herein, we described the kinetic modeling and thermodynamics of thermal inactivation of Lipase PS and Palatase, two industrial lipases of bacterial and fungal origin, respectively. MD simulations and structural analysis were also performed to explore the factors governing the thermal stability of both lipases.

### 3.1. Thermal Inactivation Analysis of Lipase PS and Palatase

**Figure 1:**
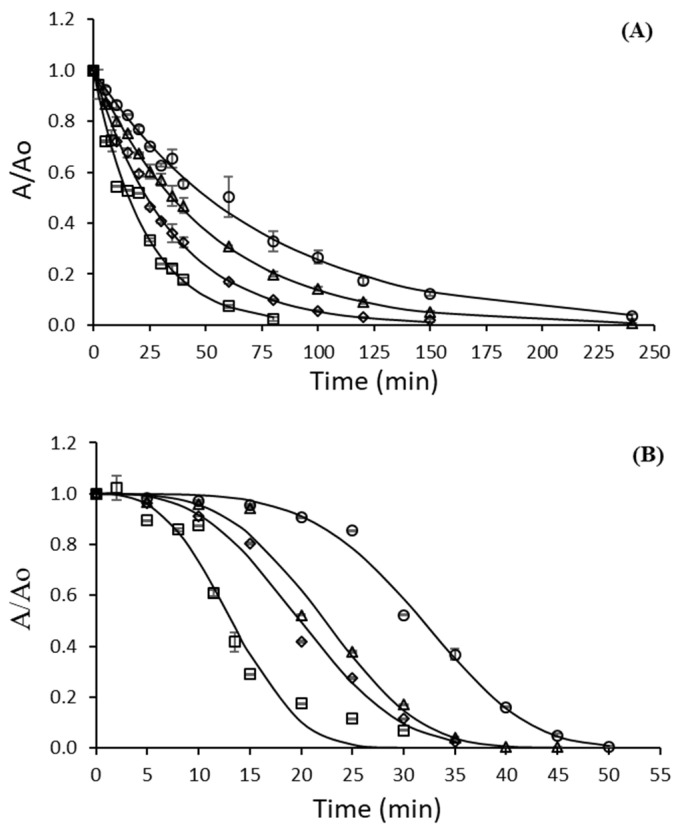
Thermal inactivation at 40 (*open circles*), 50 (*open triangle*), 60 (*open diamonds*), and 70 °C (*open square*) of lipase from *B. cepacia* (Lipase PS) (**A**) and *R. miehei* (Palatase) (**B**). Data were fitted to a first-order model (**A**) and to the Weibull model (**B**). Errors bars represent the standard deviation (SD) from three independent measurements (SD < 0.058 for Lipase PS and SD < 0.038 for Palatase).

The experimental data obtained during the thermal inactivation of lipases from *B. cepacia* (Lipase PS), and *R. miehei* (Palatase) (Figure 1) were fit using the kinetic equations included in Table 1.

For both lipases studied, a biphasic inactivation could be assumed from Figure 1 at the highest temperature (70 °C) unlike the rest of the temperatures. This biphasic behavior (described by the two-fraction model) could be due, for example, to the presence of isoenzymes of different thermostability or to the influence of pH on thermal inactivation [41]. However, the five models that suggest the presence of resistant and labile fractions inactivated by the first-order mechanism were rejected (Table 2, Equations (3)–(5), Supplementary Material Tables S1 and S2, and Figure S1). The distinct isoenzymes model results showed the same values for the inactivation rate parameters for both fractions (Palatase) or negative parameters (Lipase PS), suggesting single-step inactivation of the enzymes. Two-fraction and multi-component first-order models were refused because negative parameters were estimated. The fit of experimental data to equations that describe the series models, *nth*-order and fractional conversion (Equations (6)–(8)), did not generate coefficients of determination or parameters answers (Table 2) and was rejected. First-order and Weibull distribution models gave a good fit for the inactivation of Lipase PS (similar r^2^, χ^2^, and SEM) (Table 2). The values obtained for the criteria analyzed were very similar for both models, which did not allow for reliable choosing between them.

To determine the most suitable model for this enzyme, the dependence temperature parameters obtained for the first-order model, *k* values (Table 3), were fitted for the Arrhenius equation (Equation (11)), where *A* is the Arrhenius constant, *Ea* is the activation energy, *R* is the universal gas constant, and *T* is the absolute temperature.

(11) $$k=A\times e^{\frac{-E_{a}}{RT}}$$

*b*-Values from the Weibull distribution model were also fitted for the log-logistic equation (Equation (12)), where *T_c_* is a marker of the temperature level where the inactivation occurs at a significant rate, and *k*′ is the steepness of the *b*(T) increase once this temperature has been exceeded [30]. This equation has been used to model *b*(T) in other works successfully during thermal processes [8,42,43].

(12) $$b\left(T\right)=ln\left(1+exp\left[k^{\prime }(T-T_{c})\right]\right)$$

The r^2^ for the Arrhenius and log-logistic equations gave a good fit to the data (0.9969 and 0.9694, respectively) for Lipase PS. However, the parameter *n* = 1 obtained in the Weibull model implied a first-order behavior (Table 2, Supplementary Material Table S1). Moreover, the representation of residual activities of lipase PS versus treatment time (Figure 1A) shows a good fit of the experimental data to the lines estimated by the first model.

Unlike lipase from *B. cepacia*, the Weibull distribution model provided the best fit to the experimental data for the thermal inactivation of lipase from *R. miehei* (Table 2 and Figure 1B), with the highest values of r^2^ (0.965–0.994), the lowest of χ^2^ (0.0011–0.0026), and SEM values of 0.0017–0.0124. This model has not been applied previously to describe the thermal inactivation of lipases, although it has been proposed to explain the kinetics of thermal inactivation of other enzymes such as pectin methyl esterase [44], peroxidase [13], and proteases [8]. The Weibull rate parameter *b* (Equation (2)) emulates the thermal reaction rate, and it is characteristic of each reaction. The values of Weibull rate parameter *b* for Palatase inactivation increased, from 1.97 10^−7^ to 4.22 10^−4^ min^−n^, with increasing temperature in the interval of 40–70 °C (Table 3). It showed that lipase degradation was accelerated at higher temperatures.

**Figure 2:**
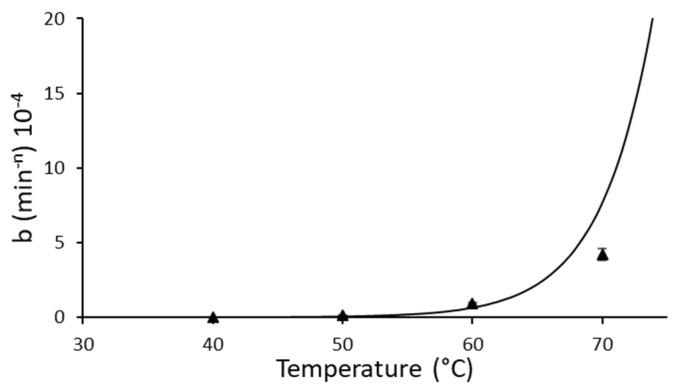
Dependence of the Weibull distribution coefficient *b* as a function of the log-logistic equation, for thermal inactivation of lipase from *R. miehei* (Palatase). The regression equation was determined as b(T) = ln (1 + exp [0.2483*(T − 98.89)]) (r^2^ = 0.9305).

The fit of b(T) to the log-logistic equation (Equation (12)) represented in Figure 2 also had a high r^2^ value (0.9305). In addition, the *n* values obtained for Palatase inactivation were higher than 1 (arranged between 2.87 and 4.36) (Table 3). The shape factor *n* > 1 implies that the semilogarithmic inactivation curve has a downward concavity [45]. This behavior has also been described for enzymatic inactivation in several works [13,46] but not for lipases. Gomes et al. [46] suggested that the enzymes are weakened by exposure to the inactivation effect and, therefore, a progressively shorter time is necessary to inactivate, i.e., the enzymatic protein will gradually become less resistant.

Limited kinetic studies of thermal inactivation of lipases from *R. miehei* and, especially, from *B. cepacia* are available in the literature. Noel and Combes [20], Yildrim et al. [21], and Carnerio et al. [22] described a series-type inactivation mechanism, first-order model, and single-step non-first order model, respectively, for lipases from *R. miehei*, and Pencreac’h et al. [19] reported a first-order model for lipase LPS AR01520 from *Pseudomonas cepacia*.

In heat processing, it is common to characterize first-order reactions in terms of half-life (*t_1/2_*) (Equation (13)) and *D*-value (Equation (14)).

(13) t12=ln(2)k

(14) $$D=\frac{2.303}{k}$$

Half-life (*t_1/2_*) is the time required to reduce 50% of the initial enzyme activity at a particular temperature. The *D*-value (decimal reduction time) is defined as the time needed for a 90% reduction in the initial activity and was calculated according to Singh and Wadhwa [47]. The rate constant increased with the higher heating temperatures, while *t_1/2_* and *D* values decreased with increasing temperature, indicating a faster inactivation at higher temperatures. It can be observed that *t_1/2_* and *D*-values for Lipase PS are 51.0 and 169 min at 40 °C, and 24.0 and 80 min at 60 °C, respectively (Table 3). In previous thermal stability studies about lipases, the half-life (*t_1/2_*) at 60 °C has been found to be equal to 5 min [20] and 0.22 min [22] for lipases from *R. miehei*, and 132 min for lipases from *P. cepacia* [19]. These variations can be attributed to several factors such as the pH and composition of the buffer during the thermal inactivation, the activity assay conditions (pH, temperature, time...), the substrate, and the differences between commercial lipases.

**Figure 3:**
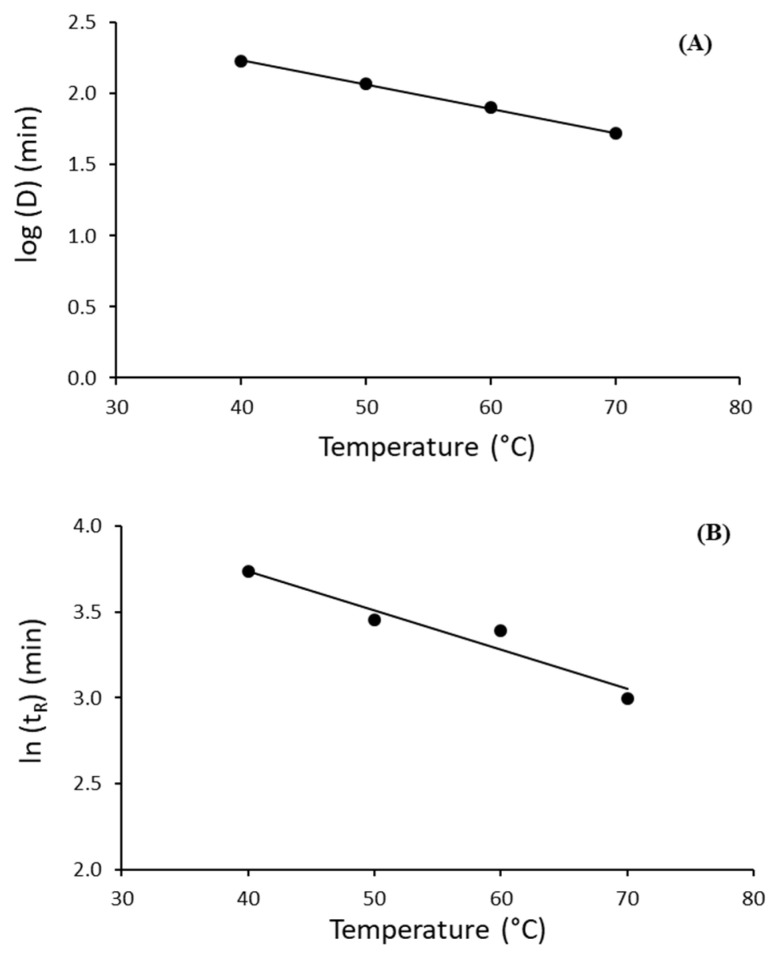
Variation of the decimal reduction time (*D*) with temperature for lipase from *B. cepacia* (Lipase PS) (**A**) and correlation between log(*t*_R_) and temperature for thermal inactivation of lipase from *R. miehei* (Palatase) (**B**). (**A**) The regression equation was determined as y = −0.0170x + 2.9121 (r^2^ = 0.9991). (**B**) The regression equation was determined as log(*t*_R_) = −0.0228T + 4.6477 (r^2^ = 0.9351).

Dependence of the *D*-value on the temperature is expressed by the z-value. The z-value was derived from *log D*-values versus temperature. The z-value indicates how many degrees of the temperature is required for decimal reduction time to be tenfold higher or lower. The effect of the temperature on *D*-values for lipase from *B. cepacia* is shown in Figure 3A, and the calculated z-value for the range of temperatures studied was 58.8 °C (Table 3).

There is no clear pattern of *n* values with temperature decrease. The Weibull model considers that the combination of *b* and *n* values results in the observed inactivation behavior. In this sense, the reliable life (*t*_R_) concept, meaning the 90% percentile of the failure time distribution, is analogous to the *D*-value (decimal reduction time) employed in the first-order analysis [48]. In our case, the term “failure” corresponds to enzymatic inactivation, *t*_R_ is calculated by Equation (15).

(15) $$t_{R}={\left(\frac{2.303}{b}\right)}^{\frac{1}{n}}$$

The *t*_R_ values for Palatase ranged from 42.0 to 20.1 min in the temperature range of 40–70 °C (Table 3). As expected, the *t*_R_ values decreased as the temperature increased indicating faster inactivation at higher temperatures. By plotting the *t*_R_ values on a log scale against the corresponding temperatures, a linear relationship (r^2^ = 0.9351) is observed (Figure 3B). The equivalent of the *z*-value (here named as *z*′-value) for the thermal inactivation of Palatase, calculated from Figure 3B, was estimated to be 43.9 °C (Table 3).

The results obtained from D and z or *t*_R_ and z’ indicated a priori a higher thermal stability of Lipase PS compared to Palatase. However, this comparison should be taken with caution since a different model was used for each lipase.

### 3.2. Thermodynamic Analysis of Lipase PS and Palatase

To determine the industrial potential of enzymes, it is essential to understand the structure–stability relationships. In this view, the estimation of thermodynamic parameters can help to understand the most probable mechanism of enzyme denaturation, which is very important in heat processes. Inactivation is known to be a process where the secondary, tertiary, or quaternary structure of a protein changes without breaking covalent bonds [49].

**Figure 4:**
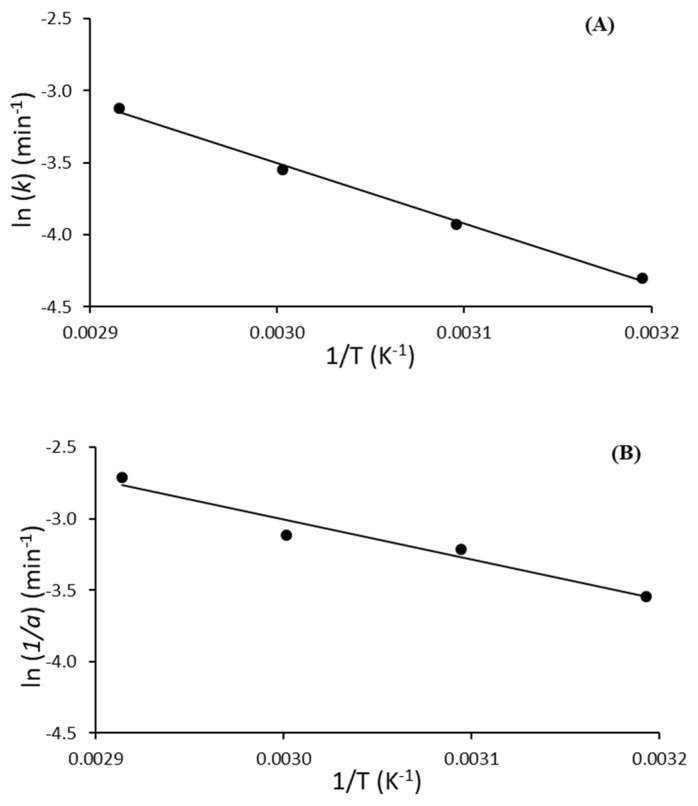
Arrhenius plot of inactivation rates of lipase from *B. cepacia* (Lipase PS) (**A**) and *R. miehei* (Palatase) (**B**). The regression equation was determined as y = −4187.4x + 9.06 (r^2^ = 0.9969) (**A**) and y = −2801.6x + 5.40 (r^2^ = 0.9531) (**B**).

The energy of activation is the minimum amount of energy required to start the deactivation process of the enzyme [50]. The activation energy (*Ea*) for lipase PS is derived from linear regression of ln(*k*) versus reciprocal temperature (1/T) (Figure 4A) and Equation (11). However, for Palatase, fitting the Weibull or any other mathematical distribution function directly to the reaction profile is merely a curve-fitting exercise that has no physical meaning [51]. Despite this, previous studies [52,53,54] have considered that the inverse of the α rate parameter (in h^−1^) versus 1/T (K^−1^) can be described with the Arrhenius equation. This parameter can be calculated from the *n* and *b* Weibull parameters as follow (Equation (16)) [55]:

(16) $$b=\frac{1}{\alpha^{n}}$$

Figure 4B represents the effect of temperature on model rate parameter α^−1^ using the Arrhenius equation. The linearity observed with r^2^ = 0.9531 confirmed that the application of the Arrhenius relationship was appropriate.

The results showed that for Lipase PS and Palatase, it was necessary to absorb 34.8 and 23.3 kJ mol^−1^, respectively, from the external medium to start its inactivation at temperatures between 40 and 70 °C (Table 4). These values indicated that higher energy was required to denature bacterial than fungal lipase, which could be attributed to a more compact structure and a more stable lid domain. This is in line with the kinetic parameters of thermal inactivation already described for both enzymes (Table 3). Higher activation energy, 64.6 kJ mol^−1^, was reported by Kumari and Gupta [56] for a thermostable lipase from *Yarrowia lipolytica*. Sonkar and Singh [17] also reported 66.9 kJ mol^−1^ (Ea) for a novel alkaline lipase from halotolerant *Bacillus gibsonii*.

The thermodynamic parameters, Gibbs free energy (ΔG^#^), enthalpy (ΔH^#^), and entropy of deactivation (ΔS^#^), were calculated as described elsewhere [12] and they are presented in Table 4. The ΔG^#^ is the energy barrier for enzyme deactivation, and it is a precise tool for evaluating the stability of the enzyme [57]. The values of ΔG^#^ for Palatase (86.0–92.1 kJ mol^−1^) were lower than that for Lipase PS (98.6–104.9 kJ mol^−1^) from 40 to 70 °C. Hence, Lipase PS showed a higher thermostability than Palatase. Results found for ΔG^#^ in this study agreed with the values of ΔG^#^ (97–99 kJ mol^−1^, from 40 to 80 °C) reported by Olusesan et al. [15] for purified lipase from *Bacillus subtilis* NS 8. Closely related results in the range of 112–121 kJ mol^−1^ were also reported for thermostable *Pseudomonas* lipases by Adams and Frawley [58], Dring and Fox [59] and Fox and Stepaniak [60]. In addition, the differences inΔG^#^ across the temperature range were about 6.5 and 7.1% for Lipase PS and Palatase, respectively (Table 4). Similar observations were found with several heat-stable structures [61,62] which may indicate a decrease in the enzyme lability to unfold as the temperature increased.

The entropy of deactivation (ΔS^#^) is correlated to the degree of variation in the local disordering between the ground state and transition state of enzyme molecules [57,63]. Thus, the enzyme and solvent disorder as well as the degrees of solvation and compactness in the protein molecular structure can be inferred through the quantitative analysis of ΔS^#^ values as reported by Ibrahim et al. [62] and Gouzi et al. [64]. Table 4 shows that the ΔS^#^ values of both lipases were negative, and the variation in ΔS^#^ was negligible across the temperature range studied. Negative values and temperature invariance of ΔS^#^ have been previously reported for several enzymes [61,62,65]. The negative value of ΔS^#^ suggested that the partially unfolded transition state was more ordered than the ground-state native structure. This can be due to water molecules forming cage-like ordered structures around non-polar amino acids upon protein unfolding [57,66,67]. The more negative ΔS^#^ for Lipase PS meant that its transition state was more ordered than that of Palatase. Thus, the inactivation of Lipase PS and Palatase suggested that the rate-limiting reaction probably involved the aggregation of partially unfolded enzyme molecules [68], possibly by the interaction of partially unfolded intermediates with exposed hydrophobic areas and/or the water ordering increases in the vicinity of nonpolar amino acids, which are exposed during unfolding [69]. Protein unfolding is accompanied by the disruption of many relative weak noncovalent bonds that form the folded structure and result in a less organized system. Other types of interactions than hydrophobic contributions play an important role in determining the thermodynamics of proteins. Specifically, the negative values of ∆S are associated with nonbonded (van der Waals) interactions and hydrogen-bond formation [70].

The degree and/or intensity of the thermal disruption of the noncovalent linkages in enzymes can be predicted through analysis of the enthalpy parameter, ΔH^#^ [62,71]. It can be seen from Table 4 that while ΔG^#^ increased with increasing temperature, ΔH^#^, like ΔS^#^, remained unchanged for each lipase. The mean values of ΔH^#^ for Lipase PS and Palatase were 32.1 and 20.6 kJ mol−^1^, respectively. The lower value of ΔH^#^ for Palatase compared to Lipase PS could mean that the fungal enzyme (Palatase) had less noncovalent bonds broken. Nevertheless, Batista et al. [72] described that the isolated values for ΔH^#^ are not suitable indicators to determine the stability of the enzyme compared to ΔG^#^.

The TΔS^#^/ΔH^#^ ratio provides a more useful measure to assess the relative contribution of enthalpy and entropy components to the enzyme inactivation [67,73]. The results obtained for this ratio (Table 4) are consistent with a reduced contribution of entropy to the inactivation process for both lipases, especially for Palatase. As the enzyme begins to unfold and/or the subunits dissociate, there will be an increase in solvent ordering around the newly exposed nonpolar aminoacidic residue side chains, enhanced by the repulsion of solvent from the nonpolar surface. This behavior will lead to a reduction in the overall entropy of the system for enzyme inactivation [67].

In summary, Lipase PS was more thermostable both enthalpically (because more heat was required to unfold native protein into the intermediate transition state) and entropically (because the transition state was more ordered). Lipase PS transition sate was harder to reach from the native folded structure than that of Palatase because it was thermodynamically unfavorable (ΔS^#^ more negative) and needed more heat to break non-covalent bonds.

### 3.3. Comparative Molecular Dynamics Simulation

One of the determining factors of the dynamic of proteins and the catalytic efficiency of enzymes is the temperature [74]. Hence, to analyze in more detail the differences observed in the thermal stability of lipases from *B. cepacia* and *R. miehei*, MD simulations and structural analyses were carried out. The selection of 313 and 343 K for MD simulations was decided to emphasize the differences between these two lipases in the range of temperatures studied.

In order to develop insights into the conformational stability and flexibility in both lipases, we performed a set of analyses such as root mean square deviation (RMSD) and root mean square fluctuation (RMSF). The dynamics of protein structure and conformation can be observed through RMSD. The RMSD is useful for the analysis of time-dependent motions of the structure and is frequently used to discern whether a structure is stable in the time scale of the simulations or if it is diverging from the initial coordinates [39]. It was found that the RMSD of the 3LIP (*B. cepacia* lipase) and 3TGL (*R. miehei* lipase) was 0.77 ± 0.26 Å and 0.84 ± 0.26 Å at 313 K, and 0.84 ± 0.24 Å and 0.92 ± 0.31 Å at 343 K, respectively. Considering that the RMSD change below 2.5 Å is an indicator of no significant conformational changes in protein structure [75], and a low RMSD indicates that the protein is stable during the simulation time [76], the RMSD results for both lipases showed that increasing the temperature to 343 K did not cause great structural differences.

**Figure 5:**
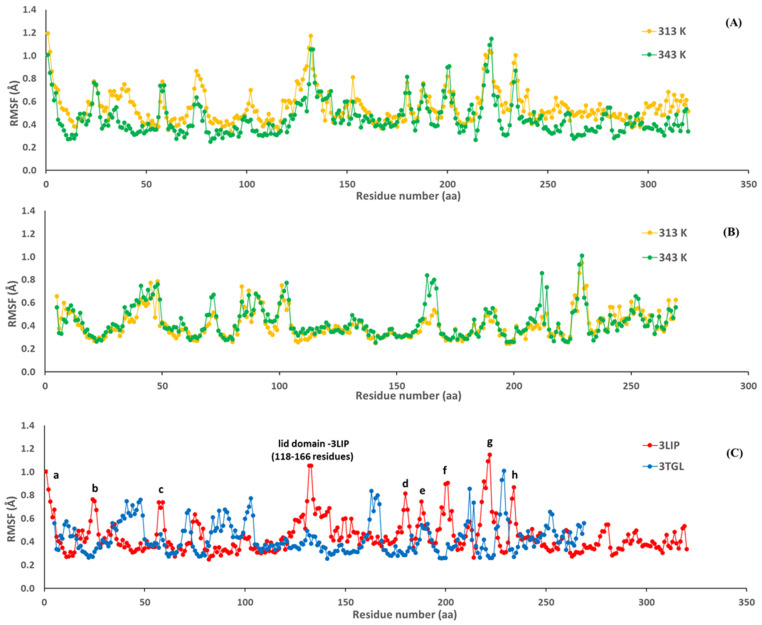
The RMSF values of Cα residues of lipase from *B. cepacia* (3LIP) and lipase from *R. miehei* (3TGL). 3LIP at 313 and 343 K (**A**), 3TGL at 313 and 343 K (**B**), and 3LIP and 3TGL at 343 K (**C**). N-terminal region (a), structural motifs such as β-hairpins (b, f, g) and β-runs (c, d, e, h).

Local plasticity of both lipases was evaluated by the RMSF of the Cα atoms. RMSF values as a function of amino-acid residues were calculated using MD simulation data of the initial structure. It can be seen from Figure 5A that increasing the temperature from 313 to 343 K did not produce higher flexibility of *B. cepacia* lipase (3LIP). However, lipase from *R. miehei* (3TGL) showed a slight increase in flexibility in some regions at 343 K (Figure 5B), which in turn, suggested a loss of structural rigidity and less compact conformation at this temperature. The average RMSF obtained for 3LIP was 0.55 ± 0.45 Å (313 K) and 0.45 ± 0.15 Å (343 K), and 0.41 ± 0.12 Å (313 K) and 0.43 ± 0.14 (343 K) for 3TGL.

The most specific feature of many lipases is the presence of a flexible lid or flap located over the active site. This dynamic domain appears to affect both the stability and the catalytic properties of lipases [5]. By superimposing the RMSF of both lipases (Figure 5C) can be observed higher local plasticity values in 3LIP compared to 3TGL at 343 K. This behavior is also observed at 313 K. For instance, Barbe et al. [77] described a particularly high mobility for lipase from the *B. cepacia* lid domain located from residues 118–166, composed by a helix-loop-helix motif including α4 and α5 helices (residues 118–127 and residues 134–150, respectively) and connected to the α6 helix (residues 160–166). This mobility is related to the conformational rearrangement of the lipase adopting the open or closed conformation. In the closed conformation, the subdomain (residues 118–159) completely obstructs the active site. The other stretches of residues in 3LIP with high levels of mobility correspond to the N-terminal region (a), and structural motifs such as β-hairpins (b, f, g) and β-runs (c, d, e, h) (Figure 5C). In contrast, the amino acids of the catalytic triad (residues 87, 286, and 264) are located in regions of very low RMSF.

**Figure 6:**
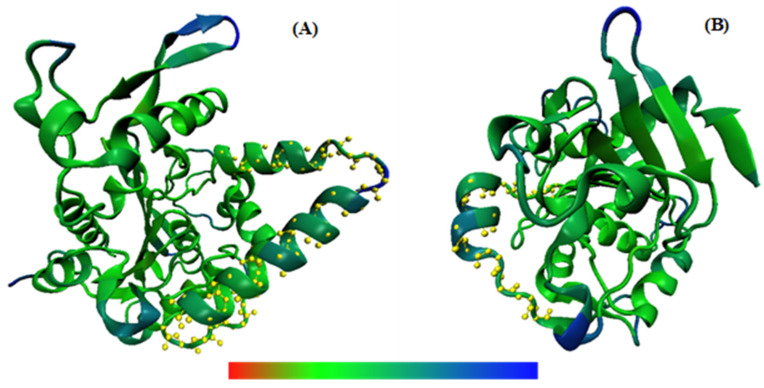
Three-dimensional backbone representations of 3LIP (from *B. cepacia*) (**A**) and 3TGL (from *R. miehei*) (**B**) structures mapped with per-residue average backbone RMSF values at a temperature of 343 K, generated using VMD. The structure color ranges from red to blue denoting that RMSF varies from the lowest to the highest values. Lid domains were highlighted by yellow balls and sticks.

Three-dimensional backbone representations corresponding to RMSF values are shown in Figure 6. It can be seen from this figure that although some features are similar for both enzymes (size, α/β hydrolase fold), 3TGL contains a small lid in the form of a helix (residues 80–95). It has been observed that the most thermostable lipases contain larger lid domains with two or more helices, whereas mesophilic lipases tend to have smaller lids in the form of a loop or a helix [5].

For these two lipases, various geometrical and structural properties, including hydrogen bonds, salt bridges, and radius of gyration (Rg) were also calculated based on the MD trajectories (Table 5). Hydrogen bonds were estimated using a geometrical criterion according to which the cut-off distance between the donor and acceptor was less than 3 Å, and the angle between the donor-hydrogen-acceptor was less than 20° and more than 50% of occupations. Salt bridges were calculated with the plugin of VMD, a distance of less than 4 Å, and more than 50% of occupations. From Table 5, it is clear that the properties such as Rg, salt bridges, and disulfide bonds were not influenced by increasing the temperature to 70 °C. The number of hydrogen bonds decreased for both lipases with an increase in the temperature from 313 to 343 K. The loss of hydrogen bonds at 70 °C for 3TGL and 3LIP was 15 and 20%, respectively (Table 5). In general, the lesser the number of hydrogen bonds, the higher the flexibility of the protein and the lower its thermostability [76]. Taking into account that lipase from *R. miehei* has a lower number of hydrogen bonds at 313 K than that of bacterial origin, the additional loss of hydrogen bonds at 343 K can be related to the slight increase in flexibility (Figure 5B) and its lower thermal stability.

On the other hand, it must be considered that the interactions between the protein and solvents also play an important role in the structural stability and function of enzymes [74]. The difference in the amino acid composition and, therefore, the variation in the surface polarity of lipases from *B. cepacia* and *R. miehei* could cause different interactions with the solvent molecules and also modify their stability.

## 4. Conclusions

Lipases are among the most important industrial enzymes. For a biocatalytic process, it is of great importance to figure out which factors govern the thermal stability of proteins. In this research, the kinetics and thermodynamics of thermal inactivation of Lipase PS from *B. cepacia* and Palatase from *R. miehei* were studied at different temperatures (40–70 °C). The kinetic modeling revealed that the Weibull distribution showed to be the best mathematical equation for heat inactivation of Palatase. To the best of our knowledge, the Weibull model has not been applied previously to describe the thermal inactivation of lipases, which means future analysis in this field should consider this approach. The temperatures required for a 90% reduction in the initial activity (z and z’ values) were 58.8 and 43.9 °C for Lipase PS and Palatase, respectively. The analysis of the T(ΔS#)/ΔH# ratio suggested that the differences in the thermal stabilities of both lipases are controlled by the enthalpic factor. The results of this study showed that the bacterial lipase was more stable to thermal inactivation than that of fungal origin. Furthermore, molecular dynamics simulation and structural analysis revealed differences in the RMSF values of Cα residues, hydrogen bonds, and lid domains that support the higher thermal stability of lipase from *B. cepacia.*

## Figures and Tables

**Table 1:** Kinetic equations used to analyze thermal inactivation of lipases.

Equation	Model	Equation ^a^	Ref.
(1)	First-order	$\frac{A}{A_{0}}=exp\;\left(-kt\right)$	[25]
(2)	Weibull distribution	$\frac{A}{A_{0}}=exp\;\left(-bt^{n}\right)$	[26]
(3)	Distinct isoenzymes	$\frac{A}{A_{0}}=A_{L}exp\;\left(-k_{L}t\right)+A_{S}exp\;\left(-k_{R}t\right)$	[12,27]
(4)	Two-fraction	$\frac{A}{A_{0}}=a\;exp\;\left(-k_{L}t\right)+\left(1-a\right)exp\;\left(-k_{R}t\right)$	[27,28]
(5)	Multi component first-order	$\frac{A}{A_{0}}=\left\{exp\;\left(-k_{1}t\right)+r\;exp\;\left(-k_{2}t\right)\right\}/\left(1+r\right)$	[29]
(6)	Series-type	$\frac{A}{A_{0}}=\alpha_{2}+\left[1+\frac{\alpha_{1}k_{1}}{k_{2}-k_{1}}-\frac{\alpha_{2}k_{2}}{k_{2}-k_{1}}\right]exp\left(-k_{1}\times t\right)-\left[\frac{\alpha_{1}k_{1}}{k_{2}-k_{1}}-\frac{\alpha_{2}k_{2}}{k_{2}-k_{1}}\right]exp\left(-k_{2}\times t\right)$	[30]
(7)	nth order decay	$\frac{A}{A_{0}}={\left\{A_{0}^{1-n}+\left(n-1\right)kt\right\}}^{1/\left(1-n\right)}$	[28]
(8)	Fractional conversion	$\frac{A}{A_{0}}=A_{r}+(A_{o}-A_{r})\;exp\;\left(-kt\right)$	[31]

^a^ *A* represents enzyme activity at time t; *A*_0_ is the initial enzyme activity; *k* is the reaction rate constant at a given temperature (s^−1^).

**Table 2:** Performance of kinetic models to describe the thermal inactivation of lipases from *B. cepacia* (Lipase PS) and *R. miehei* (Palatase).

Lipase	Model (Eq.)	r^2^	χ^2^	SEM	Remark
Lipase PS	First-order (1)	[0.971;0.996]	[0.0004;0.0030]	[0.0024;0.0170]	Accepted: high r^2^ and low SEM and χ^2^; good fit for dependence temperature parameters
	Weibull (2)	[0.973;0.996]	[0.0004;0.0325]	[0.0024;0.1819]	Rejected: *n* = 1 (first-order model)
	Distinct isoenzymes (3)	[0.974;0.997]	[0.0004;0.0068]	[0.0025;0.0383]	Rejected: negative parameter estimates
	Two-fraction (4)	[0.971;0.997]	[0.0004;0.0078]	[0.0020;0.0437]	Rejected: negative parameters estimates
	Multi component first order (5)	[0.974;0.996]	[0.0006;0.0082]	[0.0034;0.0414]	Rejected: negative parameters estimates
	Series ^a^ (6)	—	—	—	Rejected: not generate parameters answers, either coefficients of determination
	*n*th order (7)	—	—	—	Rejected: not generate parameters answers, either coefficients of determination
	Fractional conversion (8)	—	—	—	Rejected: not generate coefficients of determination
Palatase	First-order (1)	[0.687;0.833]	[0.0452;0.1601]	[0.2145;0.6537]	Rejected: low r^2^ and high SEM and χ^2^
	Weibull (2)	[0.965;0.994]	[0.0011;0.0026]	[0.0017;0.0124]	Accepted: higher r^2^ and lower SEM and χ^2^
	Distinct isoenzymes (3)	[0.749;0.872]	[0.0204;0.0425]	[0.1087;0.2144]	Rejected: equal parameter estimates; *k*_L_ = *k*_R_
	Two-fraction (4)	[0.896;0.946]	[0.0106;0.0376]	[0.0536;0.4599]	Rejected: negative parameters estimates
	Multi component first order (5)	[0.896;0.946]	[0.7850;1.6388]	[1.3653;8.2733]	Rejected: negative parameters estimates
	Series ^a^ (6)	—	—	—	Rejected: not generate coefficients of determination
	*n*th order (7)	—	—	—	Rejected: not generate parameters answers, either coefficients of determination
	Fractional conversion (8)	—	—	—	Rejected: not generate coefficients of determination

^a^ Assuming α_2_ =0, the final form of the enzyme is totally deactivated.

**Table 3:** Kinetic parameters of thermal inactivation of lipase from *B. cepacia* (Lipase PS) and *R. miehei* (Palatase).

Lipase (Model)	Temperature (°C)	r^2^	*k* (min^−1^)	*t_1/2_* (min)	D (min)	z (°C)
Lipase PS (first-order)	40	0.988	0.0136 ± 0.0003	50.97	169	58.82
	50	0.996	0.0197 ± 0.0002	35.19	117	
	60	0.998	0.0289 ± 0.0003	23.98	80	
	70	0.971	0.0440 ± 0.0014	15.75	52	
	**Temperature** **(°C)**	**r^2^**	***b*** **(min^−n^)**	***n***	***t*_R_** **(min)**	**z′** **(°C)**
Palatase (Weibull)	40	0.994	1.97 × 10^−7^ ± 1.9 × 10^−8^	4.357 ± 0.173	41.97	43.86
	50	0.987	1.54 × 10^−5^ ± 1.1 × 10^−6^	3.461 ± 0.227	31.57	
	60	0.990	9.37 × 10^−5^ ± 4.6 × 10^−6^	2.981 ± 0.158	29.70	
	70	0.965	4.22 × 10^−4^ ± 3.0 × 10^−5^	2.869 ± 0.262	20.07	

**Table 4:** Activation energy and thermodynamic parameter values of thermal inactivation of lipase from *B. cepacia* (Lipase PS) and *R. miehei* (Palatase).

Lipase	Ea (kJ mol^−1^)	Temperature (°C)	ΔH^#^ (kJ mol^−1^)	ΔG^#^ (kJ mol^−1^)	ΔS^#^ (J mol^−1^ K^−1^)	ΔS^#^/ΔH^#^
Lipase PS	34.80	40	32.20	98.57	−212.07	−2.06
		50	32.11	100.81	−212.69	−2.14
		60	32.03	102.96	−213.00	−2.21
		70	31.95	104.94	−212.80	−2.28
Palatase ^a^	23.28	40	20.68	85.97	−208.59	−3.16
		50	20.60	87.91	−208.39	−3.27
		60	20.51	90.43	−209.96	−3.41
		70	20.43	92.08	−208.90	−3.51

^a^ Apparent parameters.

**Table 5:** Structural and geometrical properties of 3LIP (from *B. cepacia*) and 3TGL (from *R. miehei*) during MD simulations.

Lipase	Temperature (K)	Rg (Å)	Hydrogen Bonds	Salt Bridges	Disulfide Bonds
3LIP	313	19.00 ± 0.08	74	2	1
	343	19.01 ± 0.05	59	2	1
3TGL	313	17.15 ± 0.05	60	6	3
	343	17.18 ± 0.06	51	6	3

## Data Availability

The data presented in this study are available on request from the corresponding author.

## Supplementary Materials

The following supporting information can be downloaded at: https://www.mdpi.com/article/10.3390/ijms23126828/s1.
