# Supplementary material for: Kinetic Modeling, Thermodynamic Approach and Molecular Dynamics Simulation of Thermal Inactivation of Lipases from Burkholderia cepacia and Rhizomucor miehei
Source: Int J Mol Sci. 2022 Jun 19;23(12):6828. doi: 10.3390/ijms23126828 (PMC9224459; doi:10.3390/ijms23126828)
Supplement: Supplementary file 1 [file ijms-23-06828-s001.zip › ijms-1740757-supplementary.pdf]

Table S1. Kinetic parameters of thermal inactivation of lipase from *B. cepacia* (Lipase PS) for Weibull (A), distinct isoenzymes (B), two-fraction (C) and multi components first order model (D)

A: Weibull model (Eq. 2)

Temperature (°C)	r^2	b (min^{-n})	n	Comment
40	0.988	0.0137±0.0016	0.997±0.027	Rejected: n=1, a particular Weibull model coincident with first order reaction
50	0.968	0.0210±0.0013	0.982±0.017	
60	0.985	0.0277±0.0019	1.013±0.020	
70	0.958	0.0542±0.0079	0.932±0.047	

B: Distinct isoenzymes model (Eq. 3)

Temperature (°C)	r^2	A_L	A_S	k_L (min^{-1})	k_S (min^{-1})	Comment
40	0.988	3.35±826	-2.36±826	0.011±0.011	0.001±0.010	Rejected: negative parameters estimates
50	0.968	8.28±9229	-2.78±9229	0.023±0.023	0.024±0.024	
60	0.985	1.03±0.130	-0.03±0.128	0.030±0.002	0.094±.313	
70	0.974	0.09±0.07	0.91±0.07	0.355±0.539	0.040±0.003	

C: Two fraction model (Eq. 4)

Temperature (°C)	r^2	a	k_L (min^{-1})	k_R (min^{-1})	Comment
40	0.988	-0.13±0.207	0.001±0.057	0.013±0.002	Rejected: negative parameters estimates
50	0.997	0.98±0.01	0.019±0.000	3.640±0.000	
60	0.996	-6.70±6E^4	0.0254±1.473	0.2580±1.303	
70	0.971	-0.11±2E^5	0.044±182.5	0.044±17.7	

D: Multi component first-order model (Eq. 5)

Temperature (°C)	r^2	r	k_1 (min^{-1})	k_2 (min^{-1})	Comment
40	0.988	-0.13±0.20	0.013±0.002	0.001±0.009	Rejected: negative parameters estimates
50	0.996	-0.01±	0.020±0.001	-0.002±0.187	
60	0.996	-0.98±6E^4	0.026±9.87	0.026±10.1	
70	0.974	13.76±15.71	0.335±0.604	0.040±0.003	

Table S2. Kinetic parameters of thermal inactivation of lipase from *R. miehei* (Palatase) for two-fraction (A) and multi components first order model (B)

A: Two fraction model (Eq. 4)

Temperature (°C)	r^2	a	k_L (min^{-1})	k_R (min^{-1})	Comment
40	0.942	-10.269±5E^4	-0.017±0.454	-0.015±0.434	Rejected: negative parameters estimates
50	0.906	-16.041±3E^4	-0.006±1.375	-0.005±1.336	
60	0.946	-11.77±1E^4	-0.014±1.047	-0.012±1.006	
70	0.896	-20.57±8E^4	-0.008±2.792	-0.009±2.724	

B: Multi component first-order model (Eq. 5)

Temperature (°C)	r^2	r	k_1 (min^{-1})	k_2 (min^{-1})	Comment
40	0.942	-0.999±4E^4	-0.016±39.303	-0.016±39.324	Rejected: negative parameters estimates
50	0.906	-0.999±2E^3	-0.006±21.732	-0.006±21.746	
60	0.946	-0.999±3E^3	-0.013±47.746	-0.013±47.783	
70	0.869	-0.999±3E^3	0.008±40.713	0.008±40.742	

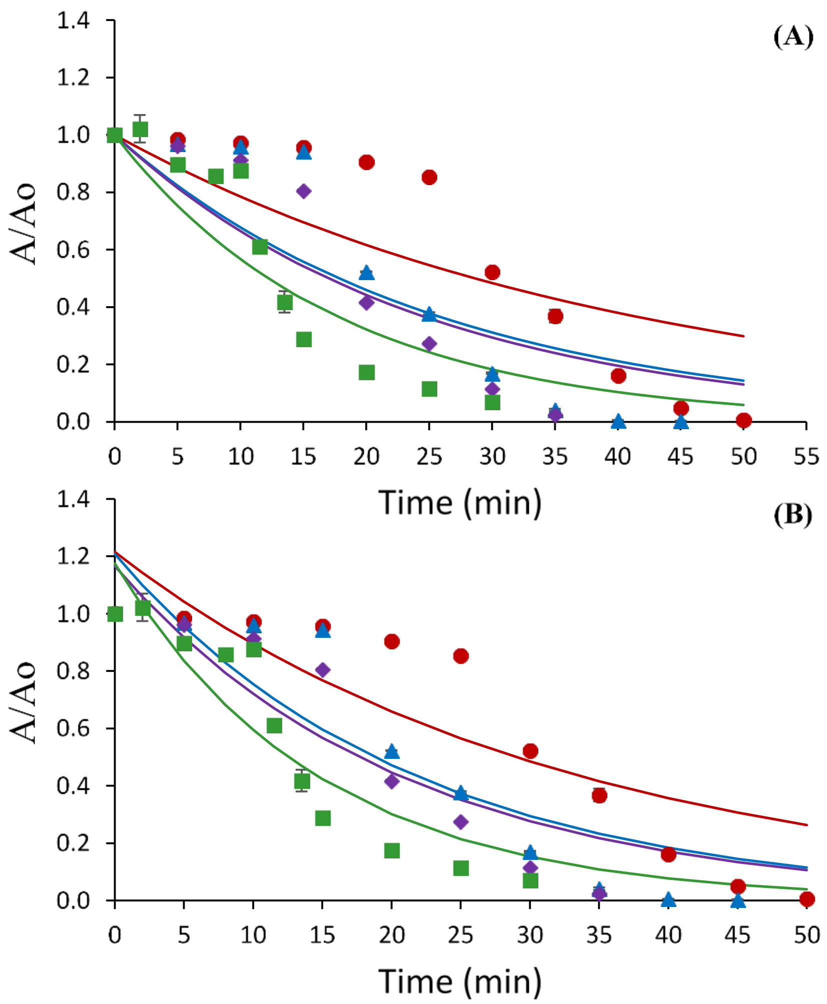

Figure S1. Thermal inactivation at 40 (red circles), 50 (blue triangle), 60 (purple diamonds) and 70 °C (green square) of lipase from *R. miehei* (Palatase). Data were fitted to a first-order model (A) and to the distinct isoenzymes model (B).
